# Workplace mistreatment and insomnia: a prospective study of child welfare workers

**DOI:** 10.1007/s00420-022-01910-3

**Published:** 2022-07-27

**Authors:** Morten Birkeland Nielsen, Sana Parveen, Live Bakke Finne

**Affiliations:** National Institute of Occupational Health, Pb 5330 Majorstuen, 0304 Oslo, Norway

**Keywords:** Sleep, Violence, Bullying, Cyber harassment, Well being

## Abstract

**Objective:**

This study examines how workplace mistreatment relates to insomnia among child welfare workers. The main aim was to determine the impact of three different forms of mistreatment, namely client perpetrated violence, cyber harassment, and colleague perpetrated bullying, on changes in levels of insomnia over time. A secondary aim was to examine whether these three forms of mistreatment represent overlapping or distinct and unique phenomena.

**Methods:**

The study was based on a probability sampled prospective survey of 424 Norwegian child welfare workers. Time lag between baseline and follow-up was six months. A confirmatory factor analysis determined the dimensionality of the indicators of mistreatment. TwoStep cluster analysis was used to examine patterns of exposure. Between and within group changes in insomnia was determined with linear regression analyses and repeated measures ANOVA. Dominance analysis was used to investigate the relative impact the predictor variables had on insomnia.

**Results:**

Client perpetrated violence and colleague perpetrated bullying were associated with increased levels of insomnia over time. Exposure to bullying was established as the most prominent predictor. Client perpetrated violence, cyber harassment, and colleague perpetrated bullying represent unique and distinct constructs. Child welfare workers mainly report exposure to one form of mistreatment rather than a combination of different types.

**Conclusions:**

Client perpetrated violence and colleague perpetrated bullying were established as risk factors for insomnia among child welfare workers. Employers and human resource personnel should prioritize developing effective primary, secondary, and tertiary strategies to prevent and handle these hazards and thereby reduce the risk of insomnia among workers.

## Introduction

The primary role of child welfare professionals is protecting children and youths from abuse, neglect, and other forms of maltreatment, and to help disadvantaged families meet the needs of their children. As this kind of work involves tight time schedules, difficult and rapid decisions, regulation of emotions, and vigilance, workers need to be mentally and physically recovered during the working day. Sleep is a necessity for recovery and without sufficient sleep, strain can accumulate and subsequently undermine health and job performance (Park and Kim 2019). Although the causes of sleep problems are complex and multifactorial, previous systematic reviews and meta-analyses have established psychosocial stress at the workplace as an important precursor to sleep problems (Linton et al. 2015; Litwiller et al. 2017). Recent evidence suggests that exposure to *workplace mistreatment* is an especially prominent work-related risk factor regarding sleep problems (Magnavita et al. 2019; Nielsen et al. 2020b). Although *workplace mistreatment* has been highlighted as a prevalent and detrimental stressor also in child welfare work (King 2021; Lamothe et al. 2021; Strolin-Goltzman et al. 2016), there is a substantial gap in the literature on how job-related stressors, including mistreatment, influence the health and health habits of child service workers (Griffiths et al. 2018; Robson et al. 2014).

Conceptually, workplace mistreatment is an umbrella term that encompasses multiple forms of physical and psychological abuse from different sources at the workplace (Asfaw et al. 2014). *Client perpetrated violence* is defined as an incident where a worker is verbally abused, threatened, or assaulted in some way by a client or client’s family member/guardian (King 2021). *Colleague perpetrated bullying* refers to a situations where an employee persistently and systematically is exposed to harassment at work from a leader or colleague and wherein this employee finds it difficult to defend him- or herself against the harassment (Einarsen 1999). *Cyber harassment* pertains to mistreatment of a worker through the use of information and communication technology (Beran and Li 2005). As cyber harassment is conducted anonymously, the perpetrator is often unknown for the target. This means that the perpetrator can be either colleagues, clients, or relatives of clients.

Although these types of mistreatment vary in severity, sources, and motives, all negatively impact employees (McCord et al. 2018). Experiencing mistreatment at work is a threat to the personal integrity of those exposed and is likely to lead to repetitive thought in the form of rumination and worry (Niven et al. 2013). Worrying and rumination have been found to be intrusive and disruptive to sleep and recovery (Berset et al. 2011). According to transactional theory of stress and coping (Lazarus and Folkman 1984), effort recovery theory (Meijman and Mulder 1998) and allostatic load (McEwen 2006), exposure to work-related stressors, such as mistreatment, requires coping efforts from the individual. Sustained efforts to cope with chronic or repeated challenges that the individual experiences as stressful will lead to physiological and psychological reactions. A potential association between workplace mistreatment and sleep is also substantiated by objective physiological evidence. Findings on disturbances in cortisol regulation show that exposure to workplace mistreatment increases levels of arousal and causes prolonged physiological activation, both of which are associated with poor sleep (Hansen et al. 2011). Compared to other stressors at the workplace, exposure to workplace mistreatment may be especially demanding as this kind of exposure is perceived as a direct threat to the target’s basic assumptions about oneself and the world (Janoff-Bulman 1992).

To determine whether workplace mistreatment is a risk factor for sleep problems among child welfare workers, the first aim of the current study is to examine the relative impact of client perpetrated violence, cyber harassment, and colleague perpetrated bullying on changes in symptoms of insomnia over time. Insomnia is included as an indicator of sleep problems as it is the most commonly reported sleep complaint in the general population with an estimated prevalence rate up to 30 percent (Ohayon 2002). A secondary aim of the study is to examine whether client perpetrated violence, cyber harassment, and colleague perpetrated bullying are overlapping or distinct and unique phenomena. Given that “workplace mistreatment” encompasses a wide array of constructs, an ongoing debate in the literature is whether mistreatment should be examined as a higher order phenomenon, or if it is more valuable to focus on the more specific second order constructs (Hershcovis 2011; Notelaers et al. 2018a). As most studies on workplace mistreatment have been restricted to examining one single construct, little is known about whether those exposed to the different forms of mistreatment perceive these as distinct or overlapping phenomena, and whether the different forms of mistreatment relate differently to individual health and well-being outcomes (Notelaers et al. 2018a; Raver and Barling 2007). That is, it is still unclear whether a proliferation of constructs is adding appreciably to our knowledge, or whether it is constraining our knowledge about workplace mistreatment (Hershcovis 2011).

## Methods

### Design and sample

The Norwegian Child Welfare Services is the public agency responsible for child protection in Norway. Each Norwegian municipality is obliged to have Child Welfare Services to secure the welfare of children and youths up to 18 years. The Child Welfare Service is responsible for the local and day-to-day implementation of the Child Welfare Act (including preventive work, investigation, support service, approval of foster families, follow-up of children placed in foster families or institutions). The data in this study were collected as part of the “Oslo Workplace Aggression Survey” (OWAS), a collaborative project between the National Institute of Occupational Health (STAMI) in Norway and The vice mayor of education and child services in Oslo municipality. All employees (*N* = 1264) working full or part time in the child welfare service in Oslo municipality were invited to participate in the survey. The baseline assessment (T1) was conducted electronically in March 2020. The follow-up (T2) was conducted in September 2020. A description of the project is provided in the project protocol (Nielsen et al. 2020a).

The project was approved by the Regional Committees for Medical and Health Research Ethics in Norway (project number 28496). In line with the General Data Protection Regulation (GDPR), the National Institute of Occupational Health acquired permission from the Norwegian Centre for Research Data (NSD; approval: 226,309) to process the personal data in this project for research purposes. The respondents had to confirm their informed consent before responding to the questionnaire. This procedure for securing informed consent was approved by the ethics committee and NSD. No personally identifiable information about respondents were available to the researchers, as data were de-identified prior to analyses.

At T1, 678 of 1265 questionnaires were returned, yielding a response proportion of 53.6 percent. At T2, 646 of 1200 invited respondents participated (response proportion 53.8%). Altogether 424 persons participated at both T1 and T2, giving a cohort response proportion of 34 percent. Descriptive statistics are presented in Table 1. The cohort consisted of 74.4 percent women and 25.6 percent men. The mean age was 39 years (SD = 10.91). A total of 82.4 percent worked in a full-time position, 10.4 percent in a part-time position, 6.6 percent were on-call staff, while 0.6 percent were on temporary leave. The latter group were excluded from the study sample. Altogether 16.6 percent of the respondents had some sort of formal leadership responsibility. Attrition analysis indicated that the T2 sample was representative of the T1 respondents on the study variables at T1: insomnia (T = 0.61: df = 559; *p* > 0.05), client perpetrated violence (T = 1.33: df = 600; *p* > 0.05), cyber harassment (T = 0.09: df = 619; *p* > 0.05), colleague perpetrated bullying (T = 0.66: df = 559; *p* > 0.05).

**Table 1:** Descriptive statistics of the cohort sample (*N* = 424)

	*N*	%	Mean	SD
*Age*			39	10.91
*Gender*				
Male	110	25.6		
Female	314	74.4		
*Employment arrangement*				
Full time	349	82.4		
Part time	44	10.4		
On-call	28	6.6		
Temporary Leave^†^	3	0.6		
*Leadership Responsibility*	70	16.6		

^†^Respondent on leave were excluded from analyses

### Instruments

Insomnia was measured with the Bergen Insomnia Scale (BIS) (Pallesen et al. 2008). BIS consists of seven items assessing difficulty to initiate and maintain sleep, and nonrestorative sleep over a period of at least three months which disrupts the person’s everyday life. Responders reported the number of days per week they had experienced the given scenarios with responses given on a ratio scale ranging from “0” to “7” days. Higher total summary scores indicate more insomnia symptoms. Previous research has shown good psychometric properties for the BIS (Pallesen et al. 2008). Cronbach’s alpha was 0.89.

Exposure to specific acts of client perpetrated physical and verbal violence and threats of violence from children, youths, and their relatives during the last six months were assessed with a 15-item behavioral experience inventory. Most items were taken from two established instruments (Barling et al. 2001; Gadegaard et al. 2018), whereas some additional items were developed for this study to capture occupation specific forms of violence. Example items are “Been threatened with a sharp object” and “Someone threatened to kill you”. Response alternatives were given on an ordinal scale using the categories “never”, “once”, “twice”, “three times”, “four times”, and “five or more times”. Cronbach’s alpha was 0.93.

*Cyber harassment* during the last six months was measured with an eight-item scale pertaining to specific harassing acts experienced through online media (internet, social media, emails etc.). Example items are “Someone spreading private information/allegations about you or your family online” and “Received messages with threats”. Response alternatives were given on an ordinal scale with the categories “Not at all”, “Seldom”, “Once in a while”, “Often”, and “Very often”. The scale was developed for the survey in cooperation with a focus group comprising representative of the child welfare profession. Hence, compared to previous cyber harassment inventories it is tailored to the working situation of child welfare workers. Cronbach’s alpha was 0.70.

*The Short Negative Acts Questionnaire* (NAQ) was used to measure perceived exposure to colleague perpetrated bullying (Einarsen et al. 2009; Notelaers et al. 2018b). The respondents were asked how often they had been exposed to bullying behavior during the last six months, with ordinal response categories on a 5-point frequency scale ranging from 1 = “never”, 2 = “occasionally”, 3 = “monthly”, 4 = “weekly” to 5 = “daily” (e.g., “If you look back over the past six months, how often did it happen that people insulted you?”). Cronbach’s alpha was 0.85.

### Control variables

Although existing evidence is inconclusive, studies have shown age differences and gender differences in occurrence (Eriksen and Einarsen 2004; Salin 2003) and outcomes (De Cuyper et al. 2009; Glambek et al. 2018; Harnois and Bastos 2018) of workplace mistreatment. There are also important gender differences in sleep. With longer sleep times, shorter sleep-onset latency and higher sleep efficiency, women have better sleep quality compared to men (Krishnan and Collop 2006). Moreover, sleep patterns tend to change with age (Ohayon et al. 2004), and working time arrangements deviating from the standard day work schedule, for instance in the form of shift work, is also a significant risk factor for insomnia (Pallesen et al. 2019). Consequently, due to their relations with mistreatment and sleep, age, gender, and working time arrangement were included as control variables in all multivariate analyses.

### Statistical analyses

Statistical analyses were performed in SPSS 26.0, MPLUS 8.4, and STATA SE 16. The dimensionality of the study variables was examined using confirmatory factor analyses in MPLUS (Muthén and Muthén 1998–2015). Due to the categorical nature of the observed indicators the Weighted Least Squares Means and Variance adjusted (WLSMV) estimator was employed to determine model fit. Being a robust estimator, the WLSMV does not require variables to be normally distributed and is therefore an adequate approach for modeling categorical or ordered data. To determine model fit, we assessed Chi-squared (CMIN) test, root mean square error of approximation (RMSEA), Tucker-Lewis Index (TLI) and comparative fit index (CFI). Values of RMSEA below 0.05 and values of CFI and TLI above 0.95 were considered indicative of a well-fitting model (Hu and Bentler 1999).

To determine patterns of workplace mistreatment, cluster analyses were conducted in SPSS using a TwoStep clustering approach (Benassi et al. 2020). This is a scalable cluster analysis algorithm designed to handle very large datasets. Capable of managing both continuous and categorical variables and/or attributes, it requires only one data pass in the procedure. In the first step of the procedure, records are pre-clustered into many small sub-clusters. In the second step, the sub-clusters are re-clustered into a desired or statistically determined number of clusters.

Linear regression and dominance analyses were used to determine the relative importance of the three indicators of workplace mistreatment on changes in levels of insomnia over time. In these analyses, we calculated mean summary variables for the indicators of mistreatment and insomnia, thus establishing average scores on a ratio scale. Descriptive analysis of insomnia at T2 showed that levels of skewness (0.70) and kurtosis (0.14) were below the thresholds for problematic distribution (Hair et al. 2006), thus indicating a close to normal distribution. In these time-lagged analyses, we regressed insomnia at T2 on the indicators of mistreatment at T1, while adjusting of levels of insomnia at T1. Hence, this is a longitudinal design that accounts for variation in the outcome variable between T1 and T2. The dominance analysis is a supplement to a multiple regression analysis which produces additive decompositions of r2 or pseudo-r2 indexes ascribing what can be interpreted as the "relative importance" of each variable or set of variables in the prediction of some outcome (Budescu 1993; Budescu and Azen 2004). The dominance analysis was carried out in STATA using the DOMIN add-on module (https://ideas.repec.org/c/boc/bocode/s457629.html).

Between and within effects regarding associations between mistreatment clusters and symptoms of insomnia across time-points were determined using repeated measures ANOVA in SPSS. The repeated measures ANOVA compares mean scores for a categorical independent variable across one or more dependent variables that are based on repeated observations.

Summary scales were calculated based on a mean-score of their respective items. Both statistical (*p* values) significance and effect sizes (strength of associations) were evaluated, with correlation coefficients (Pearson´s *r*) of about 0.1, 0.3., and 0.5 corresponding to small, medium and large effect sizes (Cohen 1988). These indices also apply for standardized beta (β) coefficients in linear regression analyses. In the repeated measures ANOVA, a partial eta squared of 0.01 indicates a small effect size, 0.06: a medium effect size, and 0.14 or higher a large effect size.

## Results

### Descriptive findings and dimensionality of constructs

At T1, 39 percent of the respondents reported exposure to at least one symptom of insomnia at least once a week during the last three months prior to the survey. Altogether 34 percent of the sample had been exposed to at least one instance of threats and/or violence at their workplace during the last six months before the survey, while 5.4 percent reported exposure to cyber harassment. Using exposure to colleague perpetrated bullying behavior at least once a month during the last six months as a criterion, 3.7 percent of the sample could be classified as targets of colleague perpetrated bullying at T1.

To determine whether the indicators of violence, online harassment and colleague perpetrated bullying are empirically different, we followed a confirmatory approach using the T1 data with five distinguishable measurement models. These were; (1) a one-dimension model with all items measuring the same latent variable (*CMIN* = 3220.88; *df* = 464*; CFI* = *0.8*8; *TLI* = 0.87; *RMSEA* = 0.097; 95% *CI RMSEA* = *0.0*94–0.100), (2) a two-dimension model with the violence items loading on one factor and cyber harassment and bullying items loading on a second factor (*CMIN* = 1444.93; *df* = 463; *CFI* = 0.96; *TLI* = 0.95; *RMSEA* = *0.0*58; 95% *CI RMSEA* = *0.0*54–0.061), (3) a two-dimension model with the violence and cyber harassment items loading on the first factor and the bullying items loading on the second factor (*CMIN* = 1829.03; *df* = 463; *CFI* = 0.94; *TLI* = 0.94; *RMSEA* = *0.0*68; 95% *CI RMSEA* = *0.0*65–0.071), (4) a two-dimension model with the violence and bullying items loading on the first factor and the cyber harassment items loading on the second factor (*CMIN* = 2485.31; *df* = 463; *CFI* = 0.91; *TLI* = 0.90; *RMSEA* = *0.0*83; 95% *CI RMSEA* = *0.0*80–0.086), and (5) a three-dimension model which included client perpetrated violence, cyber harassment, and colleague perpetrated bullying as three separate factors (*CMIN* = 1138.55; *df* = 461; *CFI* = 0.97; *TLI* = 0.97; *RMSEA* = 0.048; 95% *CI RMSEA* = *0.0*45–0.052). The fit statistics and comparisons of models indicated that the three-dimension model had the best fit to data, thus suggesting that violence, cyber harassment, and colleague perpetrated bullying represent empirically distinguishable constructs and may therefore be treated as such in analyses.

Means, standard deviations, and inter-correlations for all study variables are presented in Table 2. All three indicators of workplace mistreatment were associated with insomnia in the bivariate correlation analyses with effect sizes ranging from small to medium.

**Table 2:** Means, Standard Deviations and inter-correlations for all study variables (*N* = 424)

	Variables	Scale	M	SD	1	2	3	4	5	6	7	8
1	Sleep problems T2	0–7	2.14	1.36	–							
2	Sleep problems T1	0–7	2.21	1.57	.68^***^	–						
3	Age	–	39.10	10.91	– .13^*^	.04	–					
4	Gender	1–2	1.78	.41	.14^**^	.06	– .02	–				
5	Working time arrangement	1–2	1.37	.48	– .05	– .02	– .04	– .28***	–			
6	Client perpetrated violence	0–5	.61	.91	.19^***^	.14^**^	– .08^*^	– .31^***^	.46***	–		
7	Cyber harassment	1–5	1.09	.22	.10^*^	.15^***^	– .03	.06	– .12**	.14^**^	–	
8	Colleague perpetrated bullying	1–5	1.16	.30	.26^***^	.21^***^	– .03	.01	-.01	.17^***^	.21^***^	–

*p < .05; **p < .01, ***p < .001

Reference groups: Gender: Male; Working time arrangement: Day work

### Cluster analysis

A TwoStep cluster analysis (log-likelihood distance measure; Schwarz’s Bayesian clustering criterion) that included client perpetrated violence, cyber harassment, and colleague perpetrated bullying was conducted to investigate patterns of mistreatment in respondents at T1. The results from the main cluster analysis indicated that the baseline sample was best described by a three-cluster solution. Silhouette measure of cohesion and separation indicated good cluster quality. As displayed in Table 3, cluster 1, the “non-exposed” were characterized by lower scores on all three forms for mistreatment compared to the two other clusters. This cluster comprised 72.4 percent of the respondents. Respondents in cluster 2, totaling 17.5 percent of the sample, reported higher exposure to client perpetrated violence, but equally low scores on cyber harassment and colleague perpetrated bullying as the first clusters and where therefore labeled as “victims of violence”. The final cluster comprised 10.1 percent of the respondents. Members of this cluster reported low exposure to violence, but higher exposure to colleague perpetrated bullying and cyber harassment than the other clusters and were labeled “targets of bullying and harassment”.

**Table 3:** Scores on indicators of mistreatment and insomnia separated by clusters

	Cluster 1 “Non-exposed” (72.4%)	Cluster 2 “Victims of violence” (17.5%)	Cluster 3 “Targets of bullying and harassment” (10.1%)
Client perpetrated violence	.19 (.23)	2.14 (.95)	.71 (.61)
Cyber harassment	1.04 (.09)	1.07 (.14)	1.47 (.46)
Colleague perpetrated bullying	1.08 (.14)	1.15 (.20)	1.69 (.59)
Insomnia T1	2.05 (1.50)	2.47 (1.68)	2.84 (1.68)
Insomnia T2	1.95 (1.19)	2.48 (1.43)	2.66 (1.32)

### Associations with symptoms of insomnia

Results from the linear regression analyses of time-lagged associations between the indicators of mistreatment at the workplace and changes in levels of insomnia are presented in Table 4. After adjusting for levels of insomnia at T1 (β = 0.64; *p* < 0.001), age (β =  – 0.10; *p* < 0.05), exposure to client perpetrated violence (β = 0.10; *p* < 0.05) and colleague perpetrated bullying (β = 0.10; *p* < 0.05) emerged as significant risk factors for increased insomnia at T2. Gender, working time arrangement, and cyber harassment were not associated with changes in insomnia. The size of the beta coefficients indicated that the magnitude of the associations between violence and bullying with insomnia was small. Yet, it should be noted that this kind of effect size is to be expected when adjusting for the stability in the outcome variable in prospective studies (Zapf et al. 1996).

**Table 4:** Linear regression of prospective relationships between different types of workplace mistreatment at T1 and changes in levels of insomnia from T1 to T2 (F = 47.18; df = 6/284; *p* < .001; R2 = .50)

	Variable	B	S.E	95% CI B	β
1	Insomnia T1	.52	.04	.45 to .59	.64***
2	Age	– .01	.01	– .02 to – .00	– .10*
3	Gender	.18	.14	– .10 to.46	.06
4	Working time arrangement	– .23	.14	– .50 to .04	– .09
5	Client perpetrated violence	.15	.07	– .01 to .29	.10*
6	Cyber harassment	– .16	.24	– .63 to .30	– .03
7	Colleague perpetrated bullying	.52	.20	.06 to .85	.10*

*p < .05; **p < .01, ***p < .001

Reference groups: Gender: Male; Working time arrangement: Day work

A dominance analysis was conducted in STATA to further determine the relative impact of the indicators of workplace mistreatment on subsequent levels of insomnia. The dominance analysis showed that insomnia at T1 (rank: 1; β = 0.88) had the strongest relative relationship with insomnia at T2. Of the indicators of mistreatment, exposure to colleague perpetrated bullying (rank: 2; β = 0.08) had the strongest impact on subsequent levels of insomnia, followed by violence (rank: 3; β = 0.03). Cyber harassment (rank: 4; β = 0.01) had the lowest relative impact on insomnia.

**Fig. 1:**
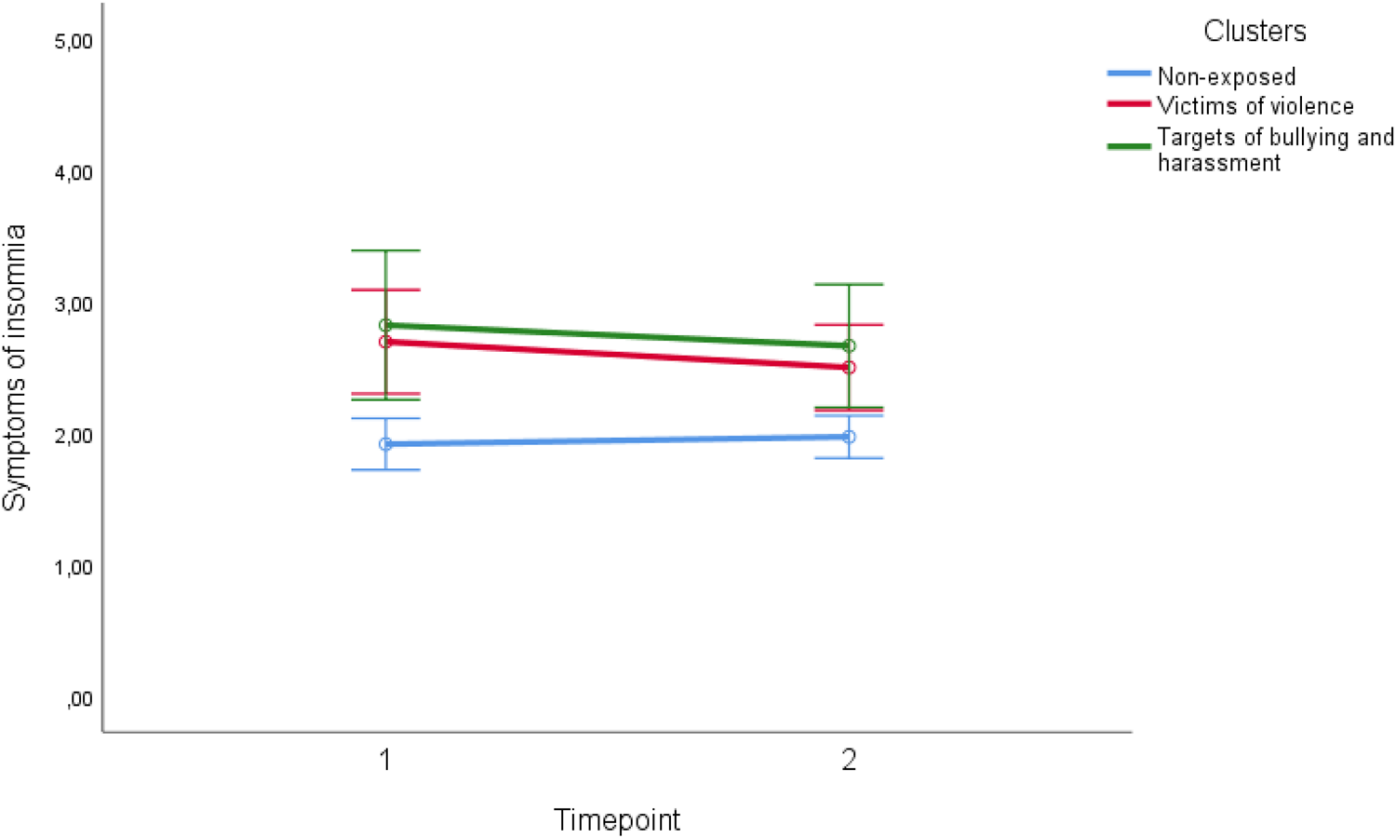
Differences between mistreatment clusters regarding symptoms of insomnia from T1 to T2 (horizontal lines represent within group changes; Error bars are 95% Confidence Intervals)

Repeated measure ANOVAs were conducted to examine differences between the mistreatment clusters on levels (between subject effects) and changes in levels (within-subject effects) of insomnia over time. Significant difference in levels of insomnia were observed between the mistreatment clusters (between subjects effect; [F(2) = 9.63, *p* < 0.001]). A Partial eta squared value of 0.06 indicated a medium difference regarding effect size. A Bonferroni post hoc test showed that respondents in the “non-exposed” cluster reported significantly lower levels of insomnia across time when compared to respondents in the “victims of violence” (mean difference: 0.65; SE = 0.19; *p* < 0.01) and the “targets of bullying and harassment” (mean difference:  – 0.80; SE = 0.26; *p* < 0.01) clusters. There were no significant differences in levels of insomnia between the “victims of violence” and “targets of bullying and harassment” clusters. Furthermore, analyses of within-subject contrasts showed no significant changes in levels of insomnia within each of the three clusters. A graphical overview of within and between effects regarding symptoms of insomnia is provided in Fig. 1.

## Discussion

Child welfare workers play a critical role in promoting child well-being and preventing abuse and neglect (King 2021). To guarantee high-standard social services to clients, it is crucial that workers are sufficiently restored and rested. Due to its regulative functions, sleep is considered as a chief determinant of physical and emotional restoration (Akerstedt 2006). Knowledge about the factors that influence sleep among child welfare workers is therefore of high importance. A main finding of the current prospective study of child service workers is that exposure to physical and psychological forms of mistreatment is associated with an increase in insomnia time, with colleague perpetrated bullying being the most prominent predictor. Our findings thereby indicate that exposure to mistreatment is a potential risk factor for sleep problems in the child welfare profession.

A secondary aim of the study was to determine whether client perpetrated violence, cyber harassment, and colleague perpetrated bullying are distinct or overlapping forms of mistreatment by those exposed. The findings showed that there is little overlap regarding exposure to these types of mistreatment. Respondents were either non-exposed, only exposed to client perpetrated violence, or only exposed to colleague perpetrated bullying (although to some extent in combination with cyber harassment).

### Explaining the findings

The prevalence rates of client perpetrated threats and violence, and colleague perpetrated bullying found in this study corresponds with the rates found among health and social workers from a recent national survey of the Norwegian working population (Bakke et al. 2021; Radey and Wilke 2021). The prevalence of cyber harassment is in line with another national survey of social workers (Fellesorganisasjonen 2017). Hence, it is likely that our sample is representative for Norwegian health and social workers in general.

The finding that exposure to workplace mistreatment is associated with reduced well-being among those exposed is in line with previous qualitative (Lamothe et al. 2018; Lamothe et al. 2021), cross-sectional (Littlechild 2005; Littlechild et al. 2016; Shin 2011) and semi-prospective (King 2021) studies on child welfare workers, as well as with findings on sleep among social workers in general (Eggli et al. 2022). However, due to the prospective design in which we adjusted for baseline levels of insomnia, this study extends most previous research by providing indications about a potential causal effect of client perpetrated violence and colleague perpetrated bullying on insomnia among child welfare workers. Compared to other well-established stressors at the workplace such as high job pace, lack of control, conflicting demands, and role ambiguity, exposure to client perpetrated violence and colleague perpetrated bullying represent direct threats to the personal integrity of those exposed (Nielsen et al. 2021). It is therefore not surprising that such forms of mistreatment are associated with increased levels of insomnia.

As discussed in the introduction, theories of effort recovery (Meijman and Mulder 1998) and allostatic load (McEwen 2006) suggest that exposure to work-related stressors will create short-term physiological and psychological reactions in employees due to the efforts needed to cope with the exposure (Eggli et al. 2022). Usually, the psychobiological system stabilizes and acute reactions to stress are reversed after having spent time off work. However, particularly stressful job incidences, such as workplace mistreatment, may elongate psychophysiological load reactions and thus threaten recovery and thereby sleep (Eggli et al. 2022). Janoff-Bulman’s (1992) trauma theory of shattered assumptions may further explain the specific impact of client perpetrated violence and colleague perpetrated bullying on the personal integrity of targets. According to this theory, individuals develop fundamental, yet unarticulated, assumptions about the world and themselves (i.e., worldviews) that allow for healthy human functioning (Edmondson et al. 2011). The most important assumptions include beliefs in a just, benevolent, predictable world in which the individual possesses competence and worth (Janoff-Bulman 1992). The primary function of these worldviews is to provide the individual with meaning, self-esteem, and the illusion of invulnerability. Being exposed to mistreatment from others challenges the targets’ basic assumptions about their own worth as well as about the world as meaningful and benevolent (Mikkelsen and Einarsen 2002). This shattering of assumptions is likely to cause persistent worrying and rumination (Mikkelsen 2001), which subsequently are risk factors for poor sleep and recovery (Berset et al. 2011). Substantiating worrying and rumination as potential mechanisms of sleep disorders, research has found that worrying is common in people suffering from sleeplessness (Pallesen et al. 2002) and is also a central end-point in line with the internalization hypothesis of insomnia (Kales et al. 1976).

The shattering of basic assumptions could also explain why exposure to colleague perpetrated bullying was more strongly related to insomnia than client perpetrated violence. While many child welfare workers are exposed to client perpetrated violence, many workers also perceive this as a normal and inevitable part of their job, or as a “call-for-help” on behalf of their clients and users (Andersson and Överlien 2018; Lamothe et al. 2018). Having this kind of mindset may provide a sense of understanding or a meaning to the exposure and is thereby likely to reduce the experience of client perpetrated violence as detrimental or problematic. In addition, from an attributional perspective, the cause of the aggression will be external and not related to worker’s personal characteristics. Taken together, this will help the worker to maintain a feeling of self-worth even when exposed to violence from clients. Colleague perpetrated bullying on the other hand, is unlikely to be perceived as normal or inevitable and will therefore be more difficult to explain for those exposed without challenging assumptions about the world and their own self-worth. Following the above line of arguments, being exposed to mistreatment from colleagues should lead to prolonged worrying and rumination and thereby also more problems with sleep.

### Implications for research and practice

The “workplace mistreatment” concept encompasses a wide array of constructs describing different negative and aggressive behaviors related to the workplace. It has been argued that this proliferation of constructs has led to a confusing situation in which many scholars are studying overlapping phenomena, but use different terminology (Spector and Fox 2005). Adding to the debate about whether workplace mistreatment should be examined as a higher order phenomenon rather than focusing in the second order constructs (Hershcovis 2011; Notelaers et al. 2018a), the findings on factor structure, clustering, and associations with outcomes in the current study indicate that child welfare workers perceive violence, cyber harassment, and bullying as distinct and unique exposures that need to be examined separately. However, it should be noted that the forms of mistreatment examined in this study originates from different sources and this is highly likely to be a strong determinant of how the exposure is perceived. Hence, regarding different forms of mistreatment from the same source (e.g., incivility and bullying from colleagues), more research is needed to determine the impact of proliferation of constructs.

This study was limited to examine the main effects of mistreatment on subsequent insomnia. Some of the most influential theories in occupational health, such as the transactional model of stress and coping (Lazarus and Folkman 1984) and the Cognitive Activation Theory of Stress (Reme et al. 2008) propose that stressor-strain relationships are determined by a range of individual and situational factors, including appraisal, coping, and social support. Therefore, in addition to replicating the main effects, future research should extend this study by also examining potential moderating and mediating factors that can contribute to explain “how and when” workplace mistreatment impact the well-being of those exposed. Furthermore, we did not examine any reverse effects of insomnia on risk of mistreatment. Although it has previously been claimed that reverse causation is unlikely in the relationship between workplace bullying and sleep (Magee et al. 2015), one should be careful dismissing sleep as a potential antecedent to mistreatment since poor sleep is likely to influence other risk factors for mistreatment such as the perceptions and behavior (Nielsen et al. 2020b). Hence, exploring the potential effects of sleep problems on risk of mistreatment is another venue for upcoming research.

The study results have consequences for workers, employers, and human resources personnel within child welfares. As our results have demonstrated that client perpetrated violence and client perpetrated bullying represent risk factors for insomnia, an up-front implication concerns the importance of developing effective primary, secondary, and tertiary strategies to prevent and handle these hazards. That is, by reducing the occurrence and impact of workplace mistreatment through primary, secondary, and tertiary interventions directed at mistreatment, this will contribute to improve the sleep quality of workers. As for primary prevention, organizational efforts to establish and maintain a strong psychosocial safety climate may be the most effective way to counteract the occurrence of workplace mistreatment (Bond et al. 2010; Law et al. 2011). To build such a climate, it is imperative that the senior management in an organization: (1) shows support and commitment to psychological health through involvement and commitment and that they take quick and decisive action to correct problems or issues that affect psychological health. (2) prioritize employee health over productivity goals. (3) Communicates with employees about issues that may affect psychological health and safety and brings these issues to the attention of the employees. (4) Involves stakeholders including employees, unions, and health and safety representatives in the occupational health and safety process, through participation and consultation (Zadow and Dollard 2016). Supporting the importance of primary prevention, findings show that prevention policies and enacted prevention behaviors at top management level, supervisor level, and among coworkers are associated with lower self-reported exposure to workplace violence and threats (Gadegaard et al. 2018).

As for secondary prevention strategies, well-developed reporting systems in combination with strong “ethical infrastructure” that enables a climate for constructive conflict management have been found to be highly important with regard to managing cases of mistreatment, and especially workplace bullying (Einarsen et al. 2017, 2018). In addition, support from supervisors seems to be beneficial following exposure to mistreatment (Lamothe et al. 2021). Finally, regarding tertiary prevention, treatment programs for social workers victimized by clients or colleagues should be of high priority. The overarching objective of such treatment programs must be to buffer and reduce the negative effects of mistreatment and to reestablish the victim’s trust and security regarding the organization, the clients, and the colleagues.

### Methodological strengths and limitations

An important strength of this study is the inclusion of a probability sample. Compared to the current response rate trend in organizational research (Stedman et al. 2019), the return rate at the T1 assessment was relatively high. Still, as there are non-responders, selection bias is likely to have occurred. There is also a risk that individuals previously exposed to workplace mistreatment may be on sick leave, have changed the line of work, or have dropped out of the workforce due to retirement or disability pension (Nielsen et al. 2017). Thus, respondents may be those with better health and resilience. We used a six months’ time-lag. Based on previous literature we argue that this time-lag is adequate for detecting the accumulated effects that result from chronic and sustained experience of stressors and strain (Ford et al. 2014). However, the time-lag between baseline and follow-up may have led to an underestimation of risk as it is likely that acute effects of mistreatment may have emerged and subsided before T1. Similarly, there may be that exposure to mistreatment has sleeper effects we were not able to capture with a six months’ time-lag. Upcoming research should therefore replicate our findings with both longer and shorter lags.

All data were collected using self-report questionnaires, which could hamper the internal validity of the findings. For instance, there is the possibility of subjective interpretations, common-method variance and response set tendencies (Spector 2006). However, as factors such as threats, colleague perpetrated bullying, and insomnia have strong subjective components and are influenced by perceptions, it is difficult to assess these phenomena using more objective methods. Several steps were taken to reduce the potential problems associated with common-method variance, including varying response anchors for different subscales, ensuring that the independent variables were presented in different sections of the survey from the dependent variable, and emphasizing to participants that their responses would be anonymous (Podsakoff et al. 2003). Although we have provided evidence for factor structure and internal consistency, it should be noted that the indicator of cyber harassment has not been validated previously. The findings on this variable should therefore be interpreted with caution.

## Conclusions

This study shows that workplace mistreatment is a widespread challenge for child welfare workers. With a prevalence rate of 34 percent, client perpetrated violence seems to be especially occurrent and our findings show that this kind of exposure is a risk factor for insomnia among workers. Colleague perpetrated bullying is also a challenge in this specific profession. Although the prevalence rates are significantly lower than those for client perpetrated violence, the impact on insomnia for those exposed seems to be even more detrimental and there is a need for preventive measures that can counteract this form of mistreatment. As for cyber harassment, we found a relatively low prevalence rate and this kind of exposure was not associated with insomnia. Taken together, the findings adds to previous evidence indicating that workplace violence and mental health need to be regularly discussed and addressed in child welfare agencies’ training, supervision, and procedures (King 2021).

## Data Availability

Data available on request from the authors.
